# Genome-wide association analysis identified molecular markers associated with important tea flavor-related metabolites

**DOI:** 10.1038/s41438-021-00477-3

**Published:** 2021-03-01

**Authors:** Kaixing Fang, Zhiqiang Xia, Hongjian Li, Xiaohui Jiang, Dandan Qin, Qiushuang Wang, Qing Wang, Chendong Pan, Bo Li, Hualing Wu

**Affiliations:** 1 Tea Research Institute, Guangdong Academy of Agricultural Sciences; Guangdong Key Laboratory of Tea Plant Resources Innovation & Utilization, Guangzhou, 510640 China; 2 Institute of Biotechnology, Chinese Academy of Tropical Agricultural Sciences, Haikou, 570100 China; 3 Hainan University, Haikou, 570228 China

**Keywords:** Plant breeding, Genetic markers

## Abstract

The characteristic secondary metabolites in tea (theanine, caffeine, and catechins) are important factors contributing to unique tea flavors. However, there has been relatively little research on molecular markers related to these metabolites. Thus, we conducted a genome-wide association analysis of the levels of these tea flavor-related metabolites in three seasons. The theanine, caffeine, and catechin levels in Population 1 comprising 191 tea plant germplasms were examined, which revealed that their heritability exceeded 0.5 in the analyzed seasons, with the following rank order (highest to lowest heritabilities): (+)-catechin > (−)-gallocatechin gallate > caffeine = (−)-epicatechin > (−)-epigallocatechin-3-gallate > theanine > (−)-epigallocatechin > (−)-epicatechin-3-gallate > catechin gallate > (+)-gallocatechin. The SNPs detected by amplified-fragment SNP and methylation sequencing divided Population 1 into three groups and seven subgroups. An association analysis yielded 307 SNP markers related to theanine, caffeine, and catechins that were common to all three seasons. Some of the markers were pleiotropic. The functional annotation of 180 key genes at the SNP loci revealed that FLS, UGT, MYB, and WD40 domain-containing proteins, as well as ATP-binding cassette transporters, may be important for catechin synthesis. KEGG and GO analyses indicated that these genes are associated with metabolic pathways and secondary metabolite biosynthesis. Moreover, in Population 2 (98 tea plant germplasm resources), 30 candidate SNPs were verified, including 17 SNPs that were significantly or extremely significantly associated with specific metabolite levels. These results will provide a foundation for future research on important flavor-related metabolites and may help accelerate the breeding of new tea varieties.

## Introduction

Tea originated in southwestern China and has been cultivated for more than 5000 years^1^. Because they are used to produce one of the three major nonalcoholic beverages worldwide (the other two being coffee and cocoa), tea plants are economically valuable crops that significantly affect society and culture. Similar to other plants, tea can synthesize its own primary metabolites, such as sugars, proteins, lipids, and nucleotides, which can then be used to synthesize secondary metabolites unique to tea plants, including caffeine (CAF), theanine (TN), and catechin components. On the basis of the number of hydroxyl groups on the B ring, the 2,3 isomer on the C ring, and whether the 3 isomer on the C ring is attached to the gallate group, the catechins can be further divided as (+)-gallocatechin (GC), (−)-epigallocatechin (EGC), (+)-catechin (C), (−)-epicatechin (EC), (−)-epigallocatechin-3-gallate (EGCG), (−)-gallocatechin gallate (GCG), (−)-epicatechin-3-gallate (ECG), and catechin gallate (CG). These characteristic substances are responsible for the unique flavors of tea^2,3^. Theanine, CAF, and catechins affect the quality of tea, but according to zoological studies, they also have diverse physiological functions. For example, TN has a calming effect and positively influences cognition, CAFs enhance bone metabolism and function as a stimulant, and catechins can protect against liver cirrhosis, lower blood pressure, and function as antimicrobial and antioxidative compounds^4^.

Because of the important effects of TN, CAF, and catechins on tea quality and their physiological functions, studies on their synthesis and regulatory pathways are crucial for the development and selection of tea plants with specific TN, CAF, and catechin levels. Previous studies on the metabolic pathways associated with these compounds involving enzymology and omics techniques (e.g., transcriptomics, proteomics, and metabolomics) have identified some genes related to their synthesis and regulation. However, limitations to the available methods for generating transgenic tea plants have made it difficult to verify and thoroughly analyze the identified functional genes in tea plants and improve tea varieties. Currently, tea plant breeders basically select new varieties through conventional breeding methods, such as by selecting elite plants from wild populations and hybrid progenies for developing new varieties^5^. Because tea is a highly heterozygous woody plant species, breeding new varieties using conventional methods is time consuming and relatively inefficient, which has resulted in a failure to satisfy the public demand for new tea products. Molecular marker-assisted selection for breeding is based on the fact that molecular markers are closely linked to target trait genes. By identifying molecular markers, the presence of genes responsible for target traits can be detected. This process is fast, accurate, and unaffected by material development. Thus, it may be applicable for selecting new tea varieties and improving tea breeding efficiency.

Molecular markers related to specific traits are currently most often obtained via genetic linkage mapping and genome-wide association analyses, with the molecular markers in tea plants primarily identified by genetic linkage mapping. To date, 19 genetic maps have been constructed for tea^6–9^, and they have been used to investigate tea yield, leaf shape, stress resistance, and secondary metabolite levels. Among these genetic maps, two are related to catechins^6,7^, one is related to CAF^8^, and one is related to TN^9^. Most of the constructed genetic maps do not include quantitative trait loci (QTLs), which may be related to the size of the maps, the accuracy of QTL mapping, and the time required for phenotypic analyses. Genome-wide association studies (GWASs) have been conducted on major food crops, but relatively few have involved tea plants. Hazra et al. completed an association analysis of 21 agronomic characteristics and the flavor-related components of 23 Dajiling tea resources, ultimately detecting 57 single-nucleotide polymorphisms (SNPs), of which 12 were related to EGCG, 8 were related to flavor, 3 were related to phenolics, 8 were related to reactive oxygen species scavenging, 6 were related to the stomatal index, 5 were related to tannins, and 7 were related to yield^10^. Wang et al. included 151 resources in an association analysis, which resulted in the identification of 26 molecular markers related to the timing of spring bud flush^11^. Zhang et al. combined RNA sequencing results for 176 resources and data regarding the levels of catechin components in a correlation analysis, which revealed that CsANR, CsF3′5′H, and CsMYB5 may influence catechin levels^12^. These studies involving natural populations examined the linkage disequilibrium between marker genes to correlate phenotypic traits with genotypes and detect marker gene loci closely related to the phenotype of interest^13,14^. Unlike QTL mapping, this method does not require the construction of a genetic map, there are no significant differences in the parental genotypes when QTLs are mapped, and multiple allelic variations in the same locus can be detected simultaneously. Additionally, genetic analyses are highly precise, even reaching the single-gene level^6^. To a certain extent, this can compensate for the disadvantage of the genetic linkage mapping method. In the current study, 289 tea plant resources, including two natural populations in a field gene bank, were analyzed. Population 1 was used for the preliminary association study to obtain candidate SNP loci associated with tea flavor-related metabolites. The detected candidate SNPs were verified in Population 2. The data presented herein may provide the foundation for future investigations on the synthesis and regulation of secondary metabolites and for the breeding of new tea varieties.

## Results

### Diversity of flavor-related metabolites

**Fig. 1:**
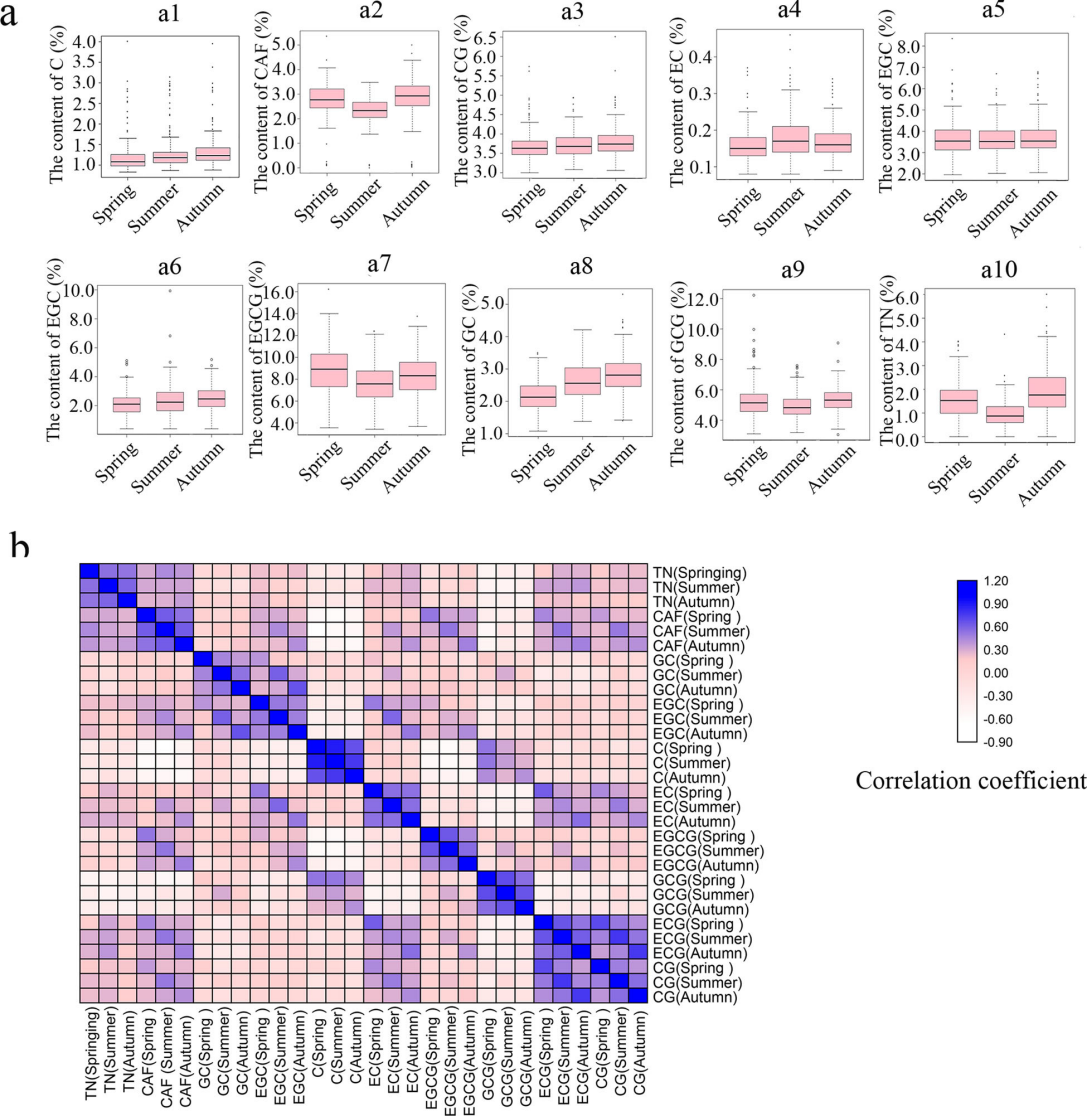
Levels of 10 metabolites in 191 germplasms in three seasons. **a** Box charts of 10 metabolites in 191 germplasms in three seasons. The seasons are presented on the abscissa, whereas the metabolite dry matter levels are presented on the ordinate. a1, C; a2, CAF; a3, CG; a4, EC; a5, ECG; a6, EGC; a7 EGCG; a8, GC; a9, GCG; a10, TN. **b** Heat map presenting the correlations among metabolite levels in different seasons. The depth of the scale color represents the strength of the correlation

In 2017, the levels of 10 flavor-related metabolites in tea buds and leaves (each bud with two leaves) from 191 tea plant resources in Population 1 (Table S1) were measured in the spring, summer, and autumn to ensure that the data were accurate. The recorded data comprised the range, mean, standard deviation, coefficient of variation, diversity index, and heritability of the metabolite dry matter content in different seasons (Table 1 and Fig. 1a). In spring, summer, and autumn, the metabolite levels changed with the seasons. Additionally, there was a significant correlation among the metabolite levels in the three seasons. The correlation was relatively strong for TN, CAF, C, EC, EGCG, GCG, ECG, and CG but relatively weak for EGC and CG. There were also correlations among the different metabolites, including between CG and ECG, between GC and EGC, and between TN and CAF (Fig. 1b and Table S2). Moreover, distinct changes in the levels of flavor-related metabolites were detected in the three seasons. For example, the TN content was significantly lower in the summer than in the spring and autumn. The CAF, EGCG, and GCG levels were slightly lower in the summer than in the spring and autumn, and there was little difference in the abundance of these three compounds between the spring and autumn. The EGC and GC levels were highest in autumn, followed by summer and spring. There was little difference in the CG, EGC, EC, and C levels in the spring, summer, and autumn. The highest and lowest coefficients of variation were calculated for the TN and CG levels, respectively. The rank order of the average coefficient of variation for the 10 analyzed metabolites in three seasons was as follows: TN > EGC > C > EC > CAF > EGCG > GC > ECG > GCG > CG. Additionally, the highest and lowest diversity indices were calculated for EGCG and C, respectively. The rank order for the average diversity index of the 10 metabolites in three seasons was as follows: EGCG > TN > EGC > GCG > GC > CG > ECG > EC > CAF > C. The heritability of the 10 metabolites ranged from 0.55 to 0.90, with the following rank order: C > GCG > CAF = EC > EGCG > TN > EGC > ECG > CG > GC.

**Table 1:** Variations in tea flavor-related metabolite levels in Population 1

Metabolite	Season	Range (%)	Mean (%)	^a^SD	^b^CV	^C^H'	Heritability
Theanine (TN)	Spring	0–4.03	1.56	0.84	0.54	2.03	0.60
	Summer	0–2.58	0.94	0.51	0.54	2.06	
	Autumn	0–6.01	1.92	1.03	0.54	2.01	
	Mean	–	1.47	0.79	0.54	2.03	
Caffeine (CAF)	Spring	0.09–5.36	2.72	0.81	0.30	1.90	0.70
	Summer	0–3.48	2.29	0.64	0.28	1.85	
	Autumn	0.03–5.0	2.84	0.81	0.29	1.83	
	Mean	–	2.62	0.75	0.29	1.86	
(+)-Gallocatechin (GC)	Spring	1.08–3.50	2.19	0.47	0.21	2.04	0.55
	Summer	1.38–4.21	2.63	0.6	0.23	2.05	
	Autumn	1.40–5.31	2.84	0.81	0.29	1.79	
	Mean	–	2.55	0.63	0.24	1.96	
(−)-Epigallocatechin (EGC)	Spring	0.40–5.11	2.11	0.81	0.39	2.02	0.59
	Summer	0.40–9.93	2.37	1.14	0.48	1.96	
	Autumn	0.40–5.18	2.48	0.93	0.38	2.07	
	Mean	–	2.32	0.96	0.42	2.02	
(+)-Catechin (C)	Spring	0.83–4.01	1.22	0.45	0.37	1.49	0.90
	Summer	0.87–3.14	1.3	0.44	0.34	1.51	
	Autumn	0.88–3.95	1.36	0.44	0.32	1.58	
	Mean	–	1.29	0.44	0.34	1.53	
(−)-Epicatechin (EC)	Spring	0.08–0.37	0.16	0.05	0.31	1.88	0.70
	Summer	0.08–0.46	0.18	0.06	0.33	1.92	
	Autumn	0.09–0.34	0.17	0.05	0.29	1.99	
	Mean	–	0.17	0.05	0.31	1.93	
(−)-Epigallocatechin-3-gallate (EGCG)	Spring	3.55–16.24	8.85	2.28	0.26	2.09	0.67
	Summer	3.42–12.40	7.47	1.87	0.25	2.08	
	Autumn	3.68–13.76	8.28	1.96	0.24	2.09	
	Mean	–	8.2	2.04	0.25	2.09	
(−)-Gallocatechin gallate (GCG)	Spring	3.10–12.22	5.27	1.16	0.22	1.87	0.75
	Summer	3.19–7.59	4.93	0.83	0.17	2.03	
	Autumn	3.05–9.08	5.35	0.83	0.17	2.02	
	Mean	–	5.18	0.94	0.19	1.97	
(−)-Epicatechin-3-gallate (ECG)	Spring	1.96–8.35	3.70	0.93	0.25	1.92	0.57
	Summer	2.02–6.70	3.62	0.73	0.20	2.00	
	Autumn	2.06–6.79	3.75	0.86	0.23	1.93	
	Mean	–	3.69	0.84	0.23	1.95	
Catechin gallate (CG)	Spring	3.00–5.74	3.70	0.42	0.11	1.93	0.56
	Summer	3.08–4.94	3.73	0.35	0.09	2.04	
	Autumn	3.06–6.51	3.82	0.43	0.11	1.89	
	Mean	–	3.75	0.4	0.1	1.95	

^a^SD, standard deviation.

^b^CV, coefficient of variation.

^c^H’, Shannon-Wiener diversity index.

### Molecular markers, group structures, and genetic relationships

Population 1, comprising 191 tea plant germplasm resources, was used to construct sequencing libraries based on the amplified-fragment single-nucleotide polymorphism and methylation (AFSM) method^15^. The sequencing resulted in 397.62 Gb of data, with an average of 2.07 Gb. The average volume was 0.67-fold that of the reference genome and 8.2-fold that of the reduced-representation genome (Table S3). On the basis of a comparison with the tea plant reference genome sequence, SNPs and insertions/deletions (indels) were identified for each sample with SAMtools and VCFtools. A total of 6,544,014 variant loci (comprising 6,152,373 SNPs and 391,641 indels) were detected. The SNPs were identified and filtered (MAF ≥ 5% and missing ≤10%) with SAMtools and VCFtools, after which 35,972 high-quality SNPs were finally obtained for further analyses. These data have been deposited in a public database (accession number: GVM000090).

**Fig. 2:**
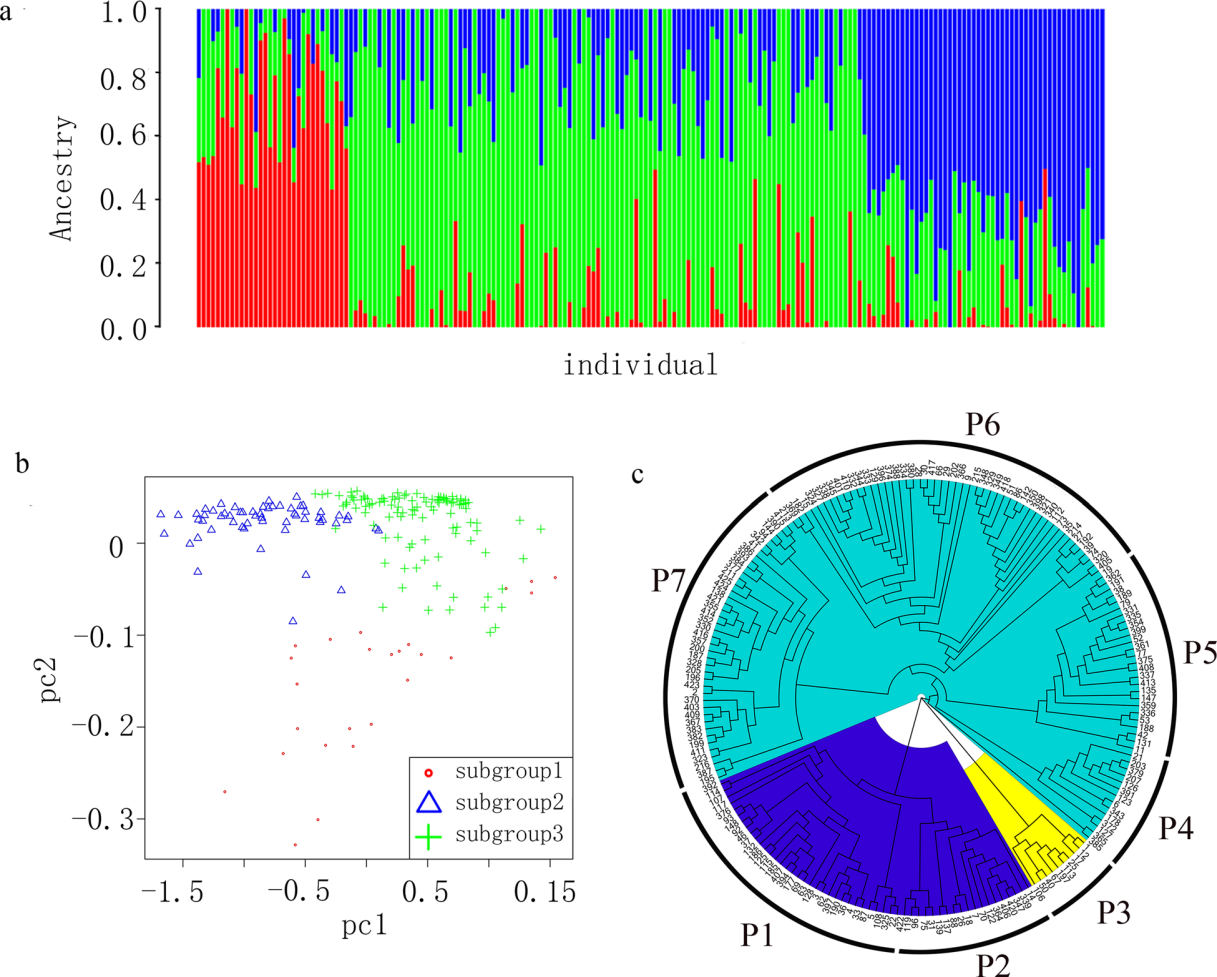
Population structures and relationships. **a** Analysis of the group structures of 191 tea resources. **b** Principal component analysis of 191 tea resources. **c** Phylogenetic tree consisting of 191 tea resources

The group structures of the obtained high-quality SNPs were analyzed with Admixture. The maximum cluster subgroup number (K) was 1–8, and the cross-validation error (CV error) of the corresponding K value was calculated. The CV error initially decreased before increasing. Because the CV error was smallest when K = 3, the group was divided into three subgroups (Fig. 2a). The results of a principal component analysis indicated that principal components PC1 and PC2 could clearly divide the 191 resources into three groups, which was consistent with the division of the group structure (Fig. 2b). A junction tree was constructed to explore the genetic relationships among the various tea germplasms. The results were consistent with those of the Admixture and principal component analyses. On the basis of the geographical distributions, biological characteristics, and genetic relationships, the 191 resources were divided into three groups. Group 1 was divided into Subgroups P1 and P2, whereas Group 3 was divided into Subgroups P4–P7 (Fig. 2c). The resources comprising highly cold-resistant shrub-like plants with small leaves were mainly distributed in Subgroup P1 and were identified as *Camellia sinensis* var. *sinensis*; these samples were collected from southeastern coastal areas, including Fujian and Zhejiang provinces. The Subgroup P2 resources were mainly Baimao tea plants (*C. sinensis* var. *pubilimba* Chang), including Lingyun Baihao tea plants from Guangxi and Lechang Baimao tea plants from northern Guangdong. Baimao tea plants have unique features, including buds and leaves that are densely covered with fuzz. By tracing the source of the analyzed germplasms, we determined that all of the Subgroup P3 resources or their parents were associated with the Yunnandaye species lineage. Subgroup P4 mainly contained the Maoye arbor population from Guangdong. These plants had large and leathery leaves. Subgroup P5 mostly comprised landraces from Guangdong, including Hakka lobular and Chaozhou Dancong resources. These landraces were collected from geographically adjacent regions, and they were characterized by small or moderately sized leaves. Subgroup P6 mainly included *C. sinensis* var. *assamica* (Masters) Kitamura arbor or semiarbor resources with large leaves as well as their natural hybrid progenies. The resources in Subgroup P7 were mixed, but most were improved cultivars, possibly because of extensive genetic recombination.

### Linkage disequilibrium of the tea plant genome

To assess the accuracy of the marker positions determined in the association study of tea plant materials, 35,972 markers were used to analyze the linkage disequilibrium between two loci. The results indicated that linkage disequilibrium decreased as the physical distance between SNPs increased^16^. In general, the decay distance of linkage disequilibrium refers to the physical distance between SNPs in the genome when *r*^2^ = 0.1 or 0.2. In the current study, when *r*^2^ = 0.1, the genetic distance between SNPs for the analyzed tea plant population was 1.8 kb (Fig. S1), which was much smaller than that for rice (123 kb), corn (30 kb), soybean (133 kb), and cassava (8 kb) but larger than that for a maize inbred line population (1.5 kb).

### SNP molecular markers associated with metabolites

**Fig. 3:**
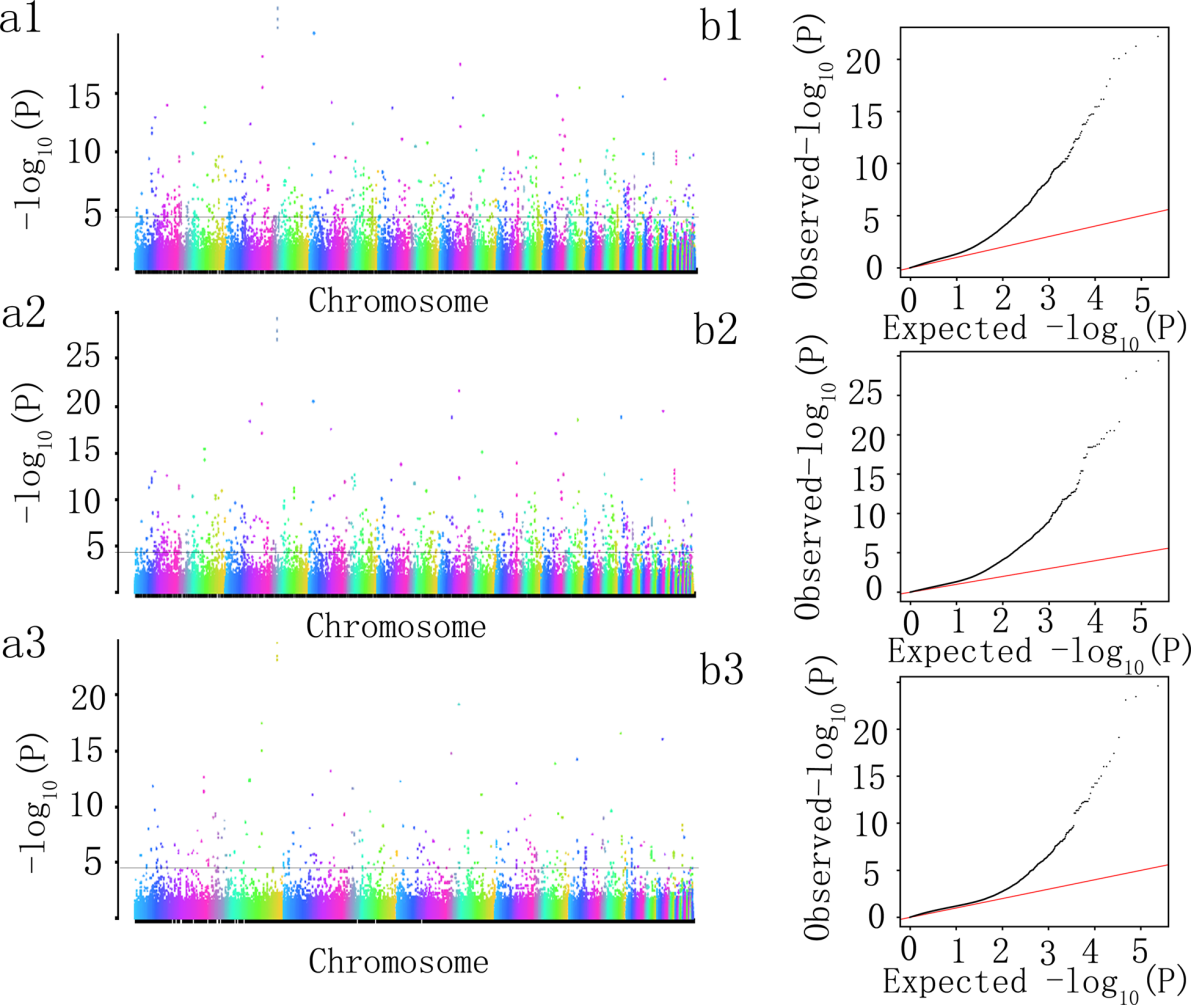
Genome-wide association analysis of C in different seasons. **a1**–**a3** Manhattan charts of the C correlation analysis in the spring, summer, and autumn. **b1**–**b3** QQ diagrams of the C correlation analysis in the spring, summer, and autumn

**Fig. 4:**
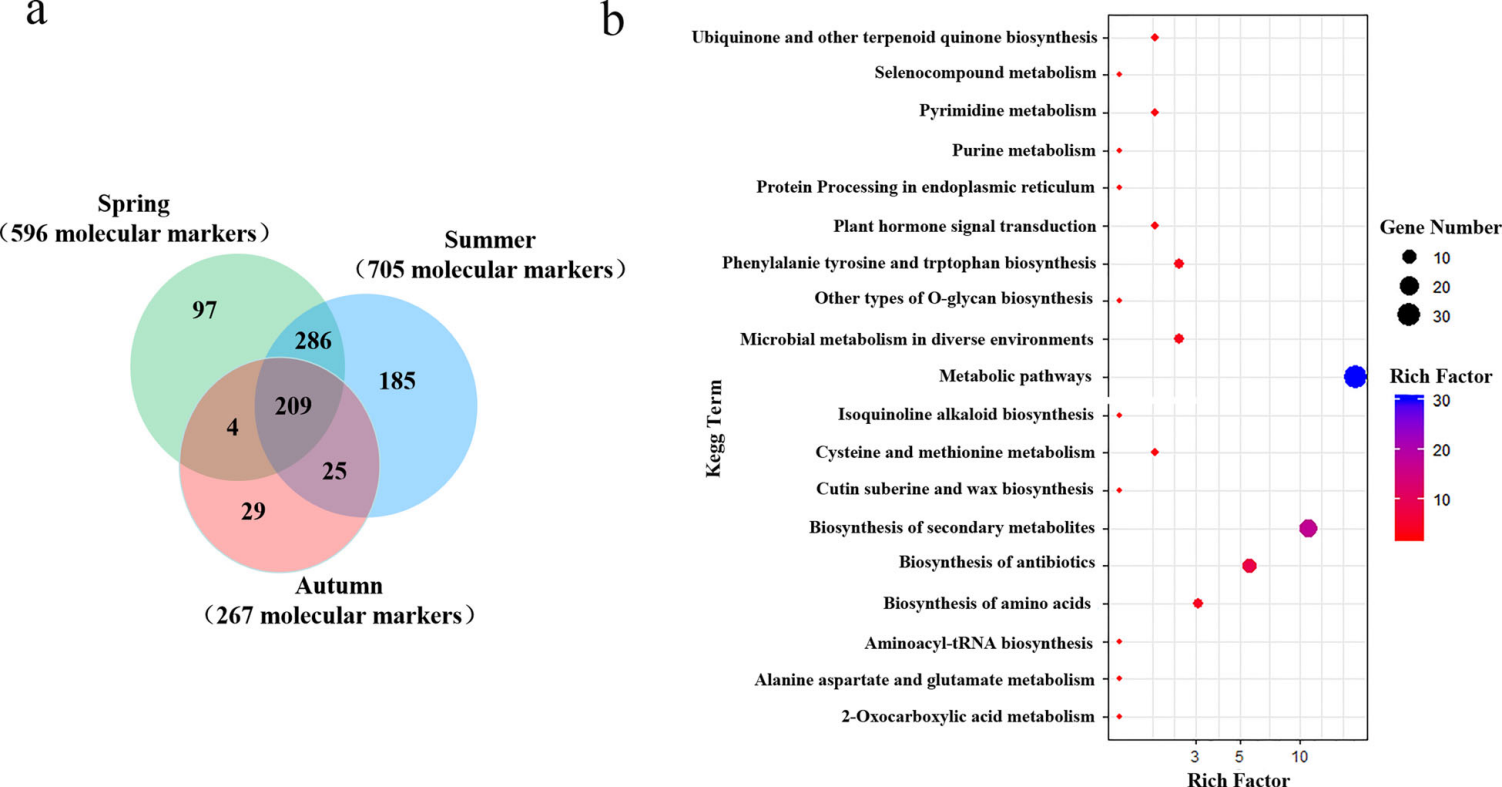
SNPs and genes identified by genome-wide association analysis. **a** Detection of catechin-related molecular markers in three seasons. **b** Top 20 enriched KEGG pathways among the genes identified by genome-wide association analysis

A mixed linear model (compressed MLM) was used to assess the correlation between 10 tea flavor-related biochemical components and 35,972 high-quality SNPs, with *P* < 2.8 × 10^−5^ applied as the threshold for screening significant loci. The number of SNPs associated with a particular metabolite varied among the seasons. To eliminate seasonal differences, we identified the significant SNPs that were detected in all three seasons and designated them candidate SNPs. Thus, 307 significantly correlated loci were identified simultaneously for all three seasons (spring, summer, and autumn) (Tables S4 and S5). With the exception of EGC and GC, significant SNPs were identified for the metabolites, with differences in the seasons in which they were identified. Regarding C, 596, 705, and 267 molecular markers were obtained for spring, summer, and autumn, respectively. Additionally, 286 SNPs were common to spring and summer, 25 SNPs were common to summer and autumn, 4 SNPs were common to spring and autumn, and 209 SNPs were common to all three seasons (Table S5, Figs. 3 and 4a). These 209 candidate SNPs were used for further analyses. The same method was used to identify 8 SNPs related to the CG content (Table S5, Figs. S2 and S3), 1 SNP related to the EC content (Table S5, Figs. S2 and S4), 51 SNPs related to the ECG content (Table S5, Figs. S2 and S5), 6 SNPs related to the EGCG content (Table S5, Figs. S2 and S6), 53 SNPs related to the GCG content (Table S4, Figs. S2 and S7), 80 SNPs related to the CAF content (Table S4, Figs. S2 and S8), and 7 SNPs related to the TN content (Table S4, Figs. S2 and S9).

Some of the molecular markers were associated with multiple metabolites (Table S4). For example, Scaffold720:596655, Scaffold720:596754, and Scaffold720:596759 were associated with five metabolites [C, CAF, EGCG, GCG, and TN]; Scaffold648:33980, Scaffold648:34046, Scaffold98:1038912, and Scaffold98:1038926 were associated with four metabolites (C, CAF, GCG, and TN); Scaffold1989:2316385 and Scaffold2922:1161116 were associated with three metabolites (C, CAF, and EGCG); Scaffold11114:266896 was associated with three metabolites (C, CAF, and GCG); and Scaffold2233:468642 was associated with two metabolites (EC and ECG). The analysis of pleiotropism indicated that C, CAF, and GCG were relatively highly correlated. Additionally, some SNP-intensive regions in the genome were related to metabolite levels. For example, six SNP loci in the 139-base sequence between Scaffold1695:2941514 and Scaffold1695:2941653 were associated with C and CAF, four SNP loci in the 46-base sequence between Scaffold1695:211063 and Scaffold1695:211109 were associated with ECG, and three SNP loci in the 104-base sequence between Scaffold720:596655 and Scaffold720:596759 were associated with C, CAF, EGCG, GCG, and TN.

### Metabolite-related genes and pathways

The genes detected in each significant locus as well as the genes located upstream or downstream of the loci were designated candidate genes, which were then functionally annotated. The genes detected in more than two seasons were used for subsequent analyses. The 180 functionally annotated genes (Table S6) were involved in 70 Kyoto Encyclopedia of Genes and Genomes (KEGG) pathways (Table S7), with the two most enriched pathways related to metabolism and the biosynthesis of secondary metabolites (Fig. 4b). The metabolic pathways were mainly related to amino acid synthesis and metabolism, lipid metabolism, and carbohydrate metabolism. The pathways related to the biosynthesis of secondary metabolites were primarily associated with flavonol biosynthesis; linoleic acid metabolism; ubiquinone and terpene biosynthesis; synthesis of tropane, piperidine, cutin, and suberine; and wax biosynthesis.

Eight metabolic pathways were associated with TN, most of which were related to the synthesis and metabolism of amino acids, such as arginine biosynthesis, tryptophan metabolism, aminoacyl-tRNA biosynthesis, and selenium complex metabolism (other amino acid metabolism). Other metabolic pathways were enriched as well, including flavonoid biosynthesis, zeatin biosynthesis, inositol phosphate metabolism, and endocytosis (Table S7). Gene ontology (GO) analysis indicated that the analyzed genes were involved in amino acid metabolism, tetrapyrrole biosynthesis, and flavonoid biosynthesis (Table S8).

Thirteen metabolic pathways were related to CAF, including biosynthesis of secondary metabolites and pathways affecting photosynthesis and photorespiration, such as ubiquinone and other terpene-quinone biosynthesis, carotenoid biosynthesis, endoplasmic reticulum protein processing, and peroxisomes. Other CAF-related metabolic pathways were associated with plant stress tolerance, namely, cutin, suberine, and wax biosynthesis, plant MAPK signaling pathway, plant hormone signaling, zeatin biosynthesis, and plant–pathogen interactions (Table S7). The GO analysis indicated that CAF was associated with photorespiration, secondary metabolism, and the cell wall (Table S8).

The 53 metabolic pathways related to C were mainly associated with the primary glucose metabolism pathway upstream of the shikimic acid pathway, the amino acid metabolic pathway related to the catechin synthesis pathway, a branch of the catechin synthesis pathway, lipid metabolism, and stress tolerance-related metabolic pathways (Table S7). The GO analysis revealed that these genes were associated with secondary metabolism, amino acid (phenylalanine, tyrosine, and tryptophan) metabolism, the cell wall, ester metabolism, and cyclic nucleotide metabolism (Table S8). The nine CG-related pathways were metabolic pathways; secondary metabolite biosynthesis; steroid biosynthesis; sesquiterpene and triterpenoid biosynthesis; antibiotic biosynthesis; cutin, suberine, and wax biosynthesis; phytohormone signaling; and endocytosis (Table S7). The GO analysis indicated that CG was associated with lipid metabolism (Table S8). The five pathways related to EC were secondary metabolite biosynthesis, linoleic acid metabolism, alpha-linolenic acid metabolism, pentose and glucuronic acid interconversion, and endocytosis (Table S7). The GO analysis revealed that EC was associated with the cell wall (Table S8). A total of 28 pathways were related to ECG, including metabolic pathways, secondary metabolite biosynthesis; amino acid (mostly including phenylalanine, tyrosine, and tryptophan) synthesis and metabolism; glycolysis; linoleic acid metabolism; pentose and glucuronic acid interconversion; and endocytosis (Table S7). The GO analysis indicated that ECG was associated with the cell wall, tetrapyrrole synthesis, glycolysis, and lipid metabolism (Table S8). We identified 26 pathways associated with GCG, including metabolic pathways, secondary metabolite biosynthesis, flavonoid biosynthesis, aminoacyl-tRNA biosynthesis, purine metabolism, ubiquitin-mediated proteolysis, zeatin biosynthesis, amino acid synthesis and metabolism, and keratin, flax, and wax biosynthesis (Table S7). The GO analysis indicated that GCG was associated with the cell wall, tetrapyrrole synthesis, lipid metabolism, flavonoids, and S-assimilation (Table S8), whereas GC was associated with ubiquitin-mediated proteolytic metabolism (Table S7). EGCG was associated with the cell wall (Table S8).

Of the 180 annotated genes, 19 were related to TN metabolism, 34 were related to CAF metabolism, 103 were related to C metabolism, 17 were related to CG metabolism, 7 were related to EC metabolism, 40 were related to ECG metabolism, 2 were related to EGCG metabolism, 4 were related to GC metabolism, and 58 were related to GCG metabolism (Table S6). Some of these genes were related to multiple metabolites. Additionally, according to the association study, most of the genes related to CAF metabolism were also associated with C metabolism, whereas the genes related to EC metabolism were also associated with ECG metabolism.

**Fig. 5:**
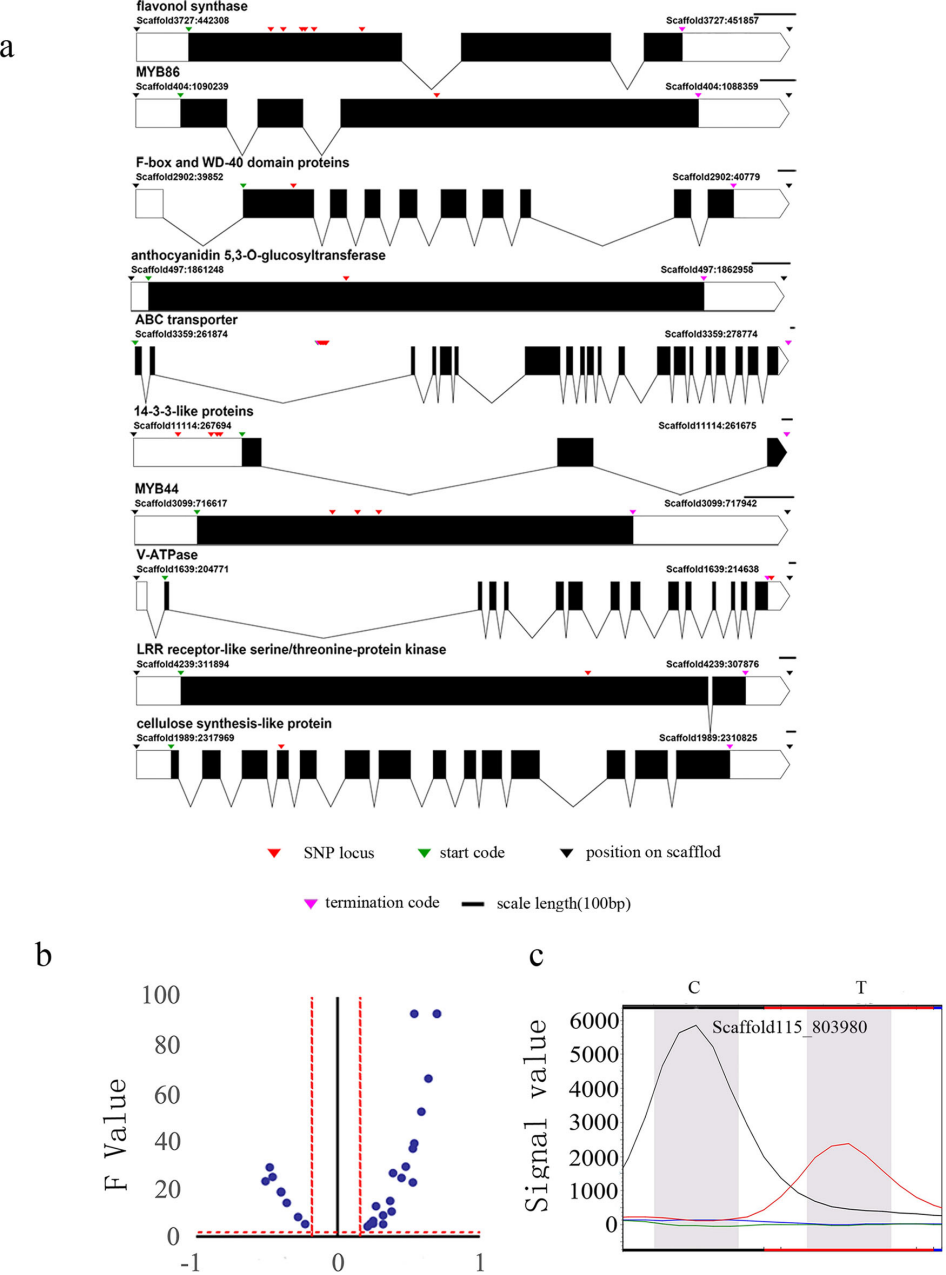
Localization and verification of candidate SNPs. **a** SNP loci in some genes identified by an association analysis. **b** F values and correlation coefficients of verified SNPs. **c** Polymorphism of validated SNPs in the analyzed resources

Some of the annotated genes were examined in previous studies and may directly or indirectly contribute to the synthesis or regulation of TN, CAF, and catechins (Table 2, Table S6, Fig. 5a). For example, flavonol synthesis is an important branch of the flavonoid pathway, and FLS is the key enzyme catalyzing flavonol production. Several studies have confirmed that catechin and anthocyanin levels in tea plants are closely related to the expression of the FLS-encoding gene^17–26^.

**Table 2:** Potential metabolite-related genes identified by association analysis

Gene ID	Annotation	Metabolites	Position	Position on the gene
TEA009498.1	14-3-3-like protein	TN	Scaffold11114:266896(266924,266980)	5′-UTR
		C	Scaffold11114:266896(266924,266980,267285)	5′-UTR
		CAF	Scaffold11114:266896(266924)	5′-UTR
		GCG	Scaffold11114:266896(266924,266980)	5′-UTR
TEA023390.1	3-ketoacyl-CoA synthase	ECG	Scaffold6390:293902	Exon
TEA016875.1	3-ketoacyl-CoA synthase	C	Scaffold2981:310444(310469,310476,310480,310486,310525,310545)	Intron
TEA013438.1	abscisate beta-glucosyltransferase	C	Scaffold2727:21888(21915,21858)	Exon
		GCG	Scaffold2727:21888(21915,21858)	Exon
		CAF	Scaffold2727:21888	Exon
TEA011416.1	ammonium transporter	C	Scaffold368:1391663	Exon
TEA005805.1	Asp aminotransferase	C	Scaffold1393:472126	Exon
TEA031504.1	Ca2^+^-transporting ATPase	C	Scaffold932:773860(773936,774028,774070)	Exon
TEA006222.1	N-terminal methyltransferase	C	Scaffold1443:95328(95340)	Exon
		CAF	Scaffold1443:95340(95328)	Exon
		GCG	Scaffold1443:95328(95340)	Exon
TEA010762.1	flavonol synthase	TN	Scaffold3727:442630(442712,442849)	Exon
		C	Scaffold3727:442660(442705,442734)	Exon
		GCG	Scaffold3727:442630(442712,442849)	Exon
TEA009057.1	glutamyl-tRNA amidotransferase	TN	Scaffold1305:184531(184781,184843)	Intron
		C	Scaffold1305:184472	Intron
		ECG	Scaffold1305:184531(184781,184843)	Intron
		GCG	Scaffold1305:184472(184531)	Intron
TEA002089.1	UDP-glucose:acetate beta-glucosyltransferase	C	Scaffold754:1721008	Exon
TEA025420.1	LRR receptor-like serine/threonine-protein kinase	C	Scaffold4239:309117	Exon
		CAF	Scaffold4239:309117	Exon
		GCG	Scaffold4239:309117	Exon
TEA002687.1	glucosyltransferase	C	Scaffold6029:364194(364274,364284)	Intron
TEA001040.1	ATP-binding cassette(ABC) transporter	TN	Scaffold3359:249334(249358,249401)	Intron
		C	Scaffold3359:266597(266602,266651,266662,266695,266729,266752,266787,266802,266818)	Intron
TEA010091.1	cellulose synthase A	C	Scaffold1989:2316385	Exon
		CAF	Scaffold1989:2316385	Exon
		EGCG	Scaffold1989:2316385	Exon
		GCG	Scaffold1989:2316385	Exon
TEA014193.1	transcription factor MYB44	C	Scaffold3099:717019(717070,717113)	Exon
		ECG	Scaffold3099:717019(717070,717113)	Exon
		GCG	Scaffold3099:717019(717070,717113)	Exon
TEA033203.1	transcription factor MYB86	C	Scaffold404:1089373	Exon
		CAF	Scaffold404:1089373	Exon
TEA004735.1	UDP-glucose:O-linked fucosebeta-1,3-glucosyltransferase	C	Scaffold786:257427	Exon
TEA014096.1	V-type H^+^-transporting ATPase subunit B	C	Scaffold1639:214361	3′-UTR
TEA027587.1	WD40 domain protein 7	C	Scaffold2902:40779	Exon
TEA023619.1	anthocyanidin 5,3-O-glucosyltransferase	GCG	Scaffold497:1861811(1861819)	Exon

There is considerable evidence indicating that most plant metabolites, including flavonoids, alkaloids, and amino acids, can be modified (glycosylated, acylated, or conjugated with other compounds) and transported to vacuoles or other subcellular chambers for temporary storage. Glycosyltransferase is involved in the glycosylation of metabolites related to the anthocyanin and catechin synthesis pathways, making it a regulator of the catechin content. The association analysis identified several glycosyltransferase-related genes that may be involved in the synthesis of catechins^20,22,23,25,27–29^. Because secondary metabolites often exert their physiological effects at sites that differ from the sites of synthesis, efficient intercellular transport, and intracellular trafficking systems are required. ATP-binding cassette (ABC) transporters are associated with the transport of alkaloids and anthocyanins^19,20,26,29^. Multiple SNPs in the ABC transporter gene sequence were related to TN and C, implying that these transporters are involved in the transport of flavor-related metabolites.

The biosynthesis of plant polyphenols is mainly regulated by the MYB-bHLH-WD40 ternary complex^17,29,30^. In this study, MYB86, MYB44, and WD40 domain-containing proteins associated with C were identified. Additionally, other genes may also influence the synthesis and regulation of TN, CAF, and catechins, including those encoding the LRR receptor-like serine/threonine-protein kinase^17,27,28^, Asp aminotransferase^17^, V-ATPase^17^, proteasome regulatory subunit^17^, Ca^2+^-transporting ATPase^18,28^, ammonium transporter^17^, cellulose synthesis-like protein^21^, 14-3-3-like proteins, N-terminal methyltransferase^30^, 3-ketoacyl-CoA synthase, glutamyl-tRNA amidotransferase, and zeatin *O*-xylosyltransferase (Table 2, Table S6). Molecular markers were distributed throughout the genes, including in exons, introns, and 5′- and 3′-untranslated regions (UTRs). The markers in some of the genes were clustered together, separated by only dozens of bases.

### Candidate SNP verification

To verify the reliability of the SNP markers, 30 candidate SNPs were validated in Population 2 comprising 98 tea plant germplasm resources (Table S9). The SNaPshot sequencing results revealed that one, two, and three alleles were amplified by 5, 23, and 2 SNP loci, respectively. The SNaPshot sequencing data for three of these 23 SNP loci with two alleles were eliminated because of the observed inconsistency with PCR amplification results, after which the remaining 20 SNP loci were verified (Tables S10 and S11, Fig. 5b, c). Correlation and significance analyses were completed based on the genotypes (Table S10) of the 20 SNP loci in 98 samples and the levels of 10 metabolites (Table S9). A total of 29 correlations were identified between the metabolites and 17 SNPs (Table 3). Ten markers were associated with C, with correlation coefficients between 0.21 and 0.70. Specifically, three markers were strongly correlated (0.6 ≤ |correlation coefficient|< 0.8) with C, five were moderately correlated (0.4 ≤|correlation coefficient|< 0.6), and two were weakly correlated (0.2 ≤|correlation coefficient|< 0.4). Additionally, eight markers were associated with CAF, with six moderately correlated and two weakly correlated. Two molecular markers were weakly associated with EC. Four molecular markers were weakly associated with ECG. One molecular marker was weakly associated with EGCG. Two molecular markers were moderately associated with GCG, and two were weakly associated.

**Table 3:** Correlation and significance analyses of candidate SNPs in Population 2

Locus	Dominance/recessiveness	Trait	Correlation coefficient	*P*-value	F-value	Significance level
Scaffold1989:2316385	Recessive mutation	C	0.45	3.16E−06	24.5	Extremely significant
Scaffold1989:2316385	Recessive mutation	CAF	−0.36	2.96E−04	14.1	Extremely significant
Scaffold3614:66549	Recessive mutation	C	0.59	1.24E−10	52.1	Extremely significant
Scaffold3614:66549	Recessive mutation	CAF	−0.48	5.45E−07	28.9	Extremely significant
Scaffold451:940283	Recessive mutation	C	0.54	8.79E−16	92.9	Extremely significant
Scaffold441:849397	Recessive mutation	GCG	0.53	6.96E−06	22.6	Extremely significant
Scaffold720:596655	Dominant mutation	C	0.39	1.42E−06	26.5	Extremely significant
Scaffold720:596655	Dominant mutation	CAF	−0.51	5.78E−06	23.1	Extremely significant
Scaffold115:803980	Recessive mutation	C	0.70	8.79E−16	92.9	Extremely significant
Scaffold115:803980	Recessive mutation	CAF	−0.40	3.66E−05	18.7	Extremely significant
Scaffold2292:1161116	Dominant mutation	EGCG	−0.28	5.13E−03	8.20	Extremely significant
Scaffold1182:2137911	Recessive mutation	C	0.21	4.25E−02	4.23	significant
Scaffold1182:2137911	Recessive mutation	CAF	−0.23	2.40E−02	5.26	significant
Scaffold1182:2137911	Recessive mutation	GCG	0.25	1.17E−02	6.61	Extremely significant
Scaffold89:479585	Recessive mutation	ECG	0.32	3.65E−03	8.89	Extremely significant
Scaffold349:3413816	Recessive mutation	C	0.48	4.78E−07	29.2	Extremely significant
Scaffold349:3413816	Recessive mutation	CAF	−0.40	4.04E−05	18.5	Extremely significant
Scaffold4239:309117	Recessive mutation	C	0.70	8.79E−16	92.9	Extremely significant
Scaffold4239:309117	Recessive mutation	CAF	−0.40	3.66E−05	18.7	Extremely significant
Scaffold4239:309117	Recessive mutation	GCG	0.53	2.67E−08	36.7	Extremely significant
Scaffold920:281727	Recessive mutation	C	0.54	7.18E−08	34.7	Extremely significant
Scaffold920:281727	Recessive mutation	CAF	−0.46	6.23E−06	23.2	Extremely significant
Scaffold3727:442660	Recessive mutation	C	0.64	1.60E−12	65.9	Extremely significant
Scaffold3180:842268	Dominant mutation	ECG	0.32	2.61E−02	5.12	significant
Scaffold1108:307422	Recessive mutation	EC	0.38	1.80E−03	10.3	Extremely significant
Scaffold1108:307422	Recessive mutation	ECG	0.25	1.86E−02	5.74	significant
Scaffold1451:851290	Recessive mutation	GCG	0.27	5.84E−04	12.7	Extremely significant
Scaffold2233:468642	Recessive mutation	EC	0.37	2.03E−04	14.9	Extremely significant
Scaffold2233:468642	Recessive mutation	ECG	0.23	2.30E−02	5.34	Significant

## Discussion

Tea plants originated in southwestern China but eventually spread elsewhere. Natural selection and artificial domestication have generated diverse tea germplasm resources, including cultivars, local populations, and wild species. Additionally, local populations and wild species are genetically highly variable and contain rare or unique secondary metabolites^30^. These resources form the basis of tea plant breeding and are critical for developing new varieties with improved traits. An examination of the levels of 10 tea flavor-related metabolites in 191 tea plant resources over three seasons revealed substantial differences. These metabolites also differed in terms of the range of variations in their levels, with relatively high coefficients of variation calculated for TN, EGC, C, and EC. Specifically, the coefficient of variation for TN was as high as 0.54, which may be related to the artificial selection of tea plant resources with specific TN levels. The coefficient of variation reflected the diversity in some metabolite levels in tea plants, and it was used as an index for selecting new varieties. Over three seasons, the metabolite levels in the analyzed tea resources underwent diverse changes, with different heritabilities. For example, the heritability of the C, GCG, CAF, EC, and EGCG levels under different environmental conditions was between 0.67 and 0.90, which is relatively high, indicating that the environmental conditions during the three seasons had a relatively minor effect on the levels of these metabolites. The heritability of the TN, EGC, ECG, CG, and GC levels was relatively low (0.55–0.6), suggesting that the abundance of these metabolites is controlled by minor genes in a complex genetic background^31^.

The genetic diversity and population structures of tea, which are essential for association studies of this important crop, are important causes of false positives in association studies^32^, necessitating the analysis of the genetic background of the population being investigated. Most of the 191 resources used in this study were from tea-growing regions in southern China. The germplasms were divided into three groups based on population genetic structures, principal component analysis, and phylogenetic relationships. A combined examination of the phylogenetic relationships, geographical distributions, and biological characteristics divided the 191 resources into seven subgroups. The tea resources in Guangdong Province mainly belong to Subgroups P4, P5, and P6, whereas most of the Yunnan and Fujian species belong to Subgroups P6 and P1, respectively. We determined that most of the tea resources in Guangdong were semiarbors with moderately sized or large leaves. The tea resources in Yunnan were mainly semiarbors with large leaves, whereas the resources in Fujian and Zhejiang were primarily shrubs with small leaves. The tea resources in Guangdong and Yunnan shared similar biological characteristics, which was consistent with the phylogenetic relationships. These results are in accordance with the findings of an earlier investigation of the genetic relationships among 450 resources in China and abroad based on EST-SSR molecular markers^33^.

The popularity of GWAS in plant genetics research increased after Klein et al. published an article describing their utility for investigating the human retina^34^. They have since been conducted to analyze important plants, including wheat, sorghum, *Arabidopsis thaliana*, maize, rapeseed, barley, and upland cotton, but not tea^35,36^. Although many candidate SNP loci have been identified by genome-wide association analyses, because of differences in sample sizes, linkage disequilibrium attenuation, number of molecular markers, population structures, and analytical models, some unrelated alleles may also be incorrectly associated with QTLs (i.e., false positives)^37^. Consequently, candidate SNPs must be validated. There are two verification methods that are commonly used. One involves the direct functional verification of candidate SNPs, whereas the other involves verification by reassociating candidate SNPs in multiple groups.

In this study, 191 tea resources were included in an association analysis of flavor-related metabolites in three seasons. The molecular markers common to all three seasons were designated candidate SNPs. Wayne diagrams indicated that the number of molecular markers varied among the three seasons, implying that the metabolite levels were regulated by internal genetic factors that were unaffected by the seasons and by changes to seasonal climates. In this study, 30 SNPs were validated in a second population, and 17 SNPs were confirmed as being significantly or extremely significantly correlated with metabolite levels. Our association analysis of TN, CAF, and catechins revealed that most of the SNP-associated functionally annotated genes are related to primary metabolic pathways, including a few that are closely related to TN, CAF, and catechin synthesis, possibly because of a common loss of heritability in the association study^38^. Factors that can decrease heritability include rare mutations^39^, structural variations^40^, epistatic interactions, and genetic and environmental interactions^41^. We also determined that some related loci have pleiotropic effects. For example, Scaffold720:596655, Scaffold720:596754, and Scaffold720:596759 were associated with five metabolites (C, CAF, EGCG, GCG, and TN). This pleiotropism might be related to the differences in specific genomic intervals of tea resources, which expanded and contracted during evolution.

To date, only 24 tea genes have been functionally verified by in vitro analyses of enzyme activities or by examining genetically transformed model plants^12,42,43^. The results of these investigations suggest that *CsANR1*, *CsANR2*, *CsLAR*, *CsF3*′*H*, *CsC4H*, *CsF3H*, *CsMYB4a*, *CsMYB5*, and *CsMYB75* might be involved in the synthesis of tea polyphenols. Additionally, *CsTCS* contributes to the synthesis of CAF^41^, whereas *CsTS1* and *CsAAPs* are involved in the synthesis and transport of TN, respectively. In the current study, 180 genes were identified in an association study and subsequently functionally annotated. These genes differ from the previously verified genes and those obtained in a recent association analysis^10,12^. However, some of the genes are located upstream or downstream of the verified genes, and some of the genes belong to the same families as these verified genes. The genes identified by our association analysis can be classified into the following four types: (1) structural genes for primary metabolism and secondary metabolite synthesis; (2) transcription factor genes regulating metabolite synthesis; (3) genes responsible for modifying and transporting metabolites; and (4) genes that coevolved with metabolite-related genes.

Some of the pathways associated with identified genes (e.g., the gene encoding the 14-3-3-like protein) affect primary metabolism, including the assimilation of carbon and nitrogen, which determines the volume of plant “sources”^44^. The absorption and efficient use of nitrogen and carbon differ among tea varieties in diverse environments, thus affecting the accumulation of downstream secondary metabolites^23,29,45–47^. Additionally, secondary metabolites are regulated at some key points of specific pathways. For example, FLS, which links flavonoids and catechins, catalyzes the hydroxylation of the C3 position in flavonoids to form various flavonols. Moreover, the MYB-bHLH-WD40 complex can regulate the expression of most flavonoid pathway genes^48^, including *CHS*, *CHI*, *ANR*, and *ANS*, to modulate catechin synthesis^17^. The modification and transport of secondary metabolites influence the synthesis of catechins. The UGT enzyme catalyzes the glycosylation of flavonoids, which stabilizes the structure of the metabolites. ABC transporters mediate the distribution of flavonoids to specific subcellular chambers where they are needed. Furthermore, some genes that coevolved with metabolite-related genes affect morphological structures, such as the cell wall, stratum corneum, and waxy layer, as well as the plant MAPK signaling pathway and plant–pathogen interactions, which may be related to the adaptative mechanism of tea plants. Xia et al. sequenced and analyzed the tea plant genome and revealed a recent genome-wide replication event that increased the copy number of genes related to tea aroma, flavor, and quality but also contributed to a substantial increase in the number of genes encoding LRR-RLK and LRR-NBS proteins, which may enable tea plants to effectively resist the adverse effects of biotic and abiotic stresses^49^. Moreover, *MYB44* was identified during our association analysis of C, ECG, and GCG. In *A. thaliana*, *AtMYB44* is important for stress responses and for enhancing disease resistance^50^.

## Materials and methods

### Plant materials

The tea plant resources used in this study were grown in the field gene bank of the Tea Research Institute of the Guangdong Academy of Agricultural Sciences (113.3 E, 24.3N). A total of 289 resources were randomly divided into two populations. Population 1 comprised 191 resources (Table S1) used for the preliminary association study, whereas Population 2 comprised 98 resources (Table S9) used for verifying the significant SNP loci. These resources were mainly collected from approximately 10 provinces in China, but a few were foreign resources. Some of these resources were bred varieties collected from various provinces, and some were derived from natural hybridization, with populations collected from different regions. The selected resources were randomly distributed in the field gene bank. They were planted in separate rows. Each row was 4 m long, with a row spacing of 1.5 m and a plant spacing of 20 cm. The field gene bank was managed according to local tea cultivation practices. For the 191 resources in Population 1, tea buds and leaves (each bud with two leaves) were collected on March 15 (spring), June 25 (summer), and September 28 (autumn), 2017. Regarding the 98 resources in Population 2, the buds and leaves were collected on May 1, 2018. For all germplasm resources, three biological replicates were collected from the field gene bank in each season. Samples were prepared according to the national standards of China^51^. Replicates were analyzed separately, and the data are presented in Tables S1 and S9.

### Phenotypic data analysis

The 10 metabolites associated with tea flavor were detected by high-performance liquid chromatography. Specifically, TN was analyzed according to Agilent’s precolumn derivatization method, whereas CAF, GC, EGC, C, EC, EGCG, GCG, ECG, and CG were examined according to the national standard method. The range, mean, standard deviation, and coefficient of variation for each metabolite were analyzed with SPSS software. Quantitative data for the metabolites were divided into 10 grades with standard deviations of 0.5 and used to calculate the Shannon-Wiener diversity index for the metabolites. The best linear unbiased prediction method was applied to estimate the breeding value with a 1-year multipoint model while estimating the generalized heritability.

### Genotype analysis

Total DNA was extracted from the sprouts of the 191 tea plant resources in Population 1 according to the CTAB method. The A260/A280 of each DNA sample was confirmed to be between 1.8 and 2.0, and the concentration was greater than 100 µg/µl. The extracted DNA samples were used to construct libraries based on the AFSM method^15^ for subsequent high-throughput sequencing with the Illumina HiSeq 2000 apparatus. The raw sequencing data were optimized with a Perl script to remove low-quality reads. The remaining clean reads were assigned to each individual based on the designed tags. Reads were compared with the *C. sinensis* var. *sinensis* Shuchazao cultivar genome sequence^52^, after which SNPs were identified and filtered with SAMtools and VCFtools. The conditions for filtering the SNPs were as follows: MAF ≥ 5% and missing ≤10%. The datasets described herein were submitted to the Genome Variation Map (GVM) database of the Big Data Center, Beijing Institute of Genomics, Chinese Academy of Science.

### Population structure, kinship, and linkage disequilibrium analysis

The.ped and.map files of the SNP genotype data were converted to.bed files with Plink and used as input files. The GCTA program was used to estimate the genetic relationships between individuals. A principal component analysis was also completed, with the scatter plot drawn with R software. On the basis of the genetic distance matrix, the PHYLIP neighbor-joining function was applied, and the default rootless tree algorithm was used to construct a phylogenetic tree, which was drawn with Figtree software. Moreover, Plink was used to identify SNPs with high linkage disequilibrium, extract SNPs with relatively low linkage disequilibrium, and generate a binary.bed file. The subgroup number K was set between 1 and 10, and the optimal K value was determined according to the CV error. A genetic structure matrix was drawn based on the genetic component coefficient (Q) of each material in each subgroup. The filtered SNPs were used to calculate the *r*^2^ value between two molecular markers. A scatter plot of the smooth curve was drawn with the distance as the abscissa and the average *r*^2^ values as the ordinate. The abscissa of the intersection of the curve and the straight line *r*^2^ = 0.1 represented the attenuation distance of the linkage disequilibrium.

### Association study

The filtered 35,972 high-quality SNPs and indels were used for the GWAS of 10 tea flavor-related metabolites over three seasons. The mixed linear model in the TASSEL program was used, with the genetic component coefficient Q value and the kinship K value applied as covariates. The genotype data were used to analyze the correlation between metabolite levels and markers. The Manhattan map and the quantile-quantile scatter plot were generated with the qqman package of R with the −log_10_(*P*) observed and expected values for each SNP locus. The significance threshold was set at 1/n, where n is the total number of markers.

### Candidate gene identification and pathway analysis

The significantly associated SNP loci common to all three seasons were functionally annotated. Additionally, the genes detected at each locus or upstream or downstream of the loci were designated candidate genes. If a locus was between two genes, the nearest gene was identified as a candidate gene. The candidate genes were annotated based on the tea plant genome annotation file^50^. The KEGG pathway database (http://www.genome.jp/kegg) and the Gene Ontology Consortium database (http://geneontology.org) were then used to assign metabolic pathways to the candidate genes.

### Candidate SNP marker verification

Thirty significantly associated loci were selected as candidate SNPs and verified in Population 2. Buds and leaves (each bud with two leaves) were collected from the tea plant resources in Population 2 for an analysis of the levels of the 10 tea flavor-related metabolites. Total DNA was extracted from the 98 samples in Population 2. The PCR amplification primers (Table S12) and the single-base extension primers (Table S13) were designed according to the sequences upstream and downstream of the 30 SNP loci. The PCR-amplified products were processed with the SAP digestion system. The SNaPshot reaction was followed by sequencing. The genotypes of the 30 SNPs corresponding to each sample were counted. Correlation and significance analyses involving the data for the 10 metabolites were performed with SPSS.

## Supplementary information

Table S1

Table S2

Table S3

Table S4

Table S5

Table S6

Table S7

Table S8

Table S9

Table S10

Table S11

Table S12

Table S13

## Supplementary information

**Supplementary information** The online version contains supplementary material available at 10.1038/s41438-021-00477-3.
